# Network regression analysis in transcriptome-wide association studies

**DOI:** 10.1186/s12864-022-08809-w

**Published:** 2022-08-06

**Authors:** Xiuyuan Jin, Liye Zhang, Jiadong Ji, Tao Ju, Jinghua Zhao, Zhongshang Yuan

**Affiliations:** 1 Department of Biostatistics, School of Public Health, Cheeloo College of Medicine, Shandong University, Jinan, 250012 Shandong China; 2 Institute for Medical Dataology, Shandong University, Jinan, 250003 Shandong China; 3 Institute for Financial Studies, Shandong University, Jinan, 250100 Shandong China; 4 Department of Public Health and Primary Care, Cardiovascular Epidemiology Unit, University of Cambridge, Cambridge, UK

**Keywords:** TWAS, Biological networks, Dirichlet process regression, Pointwise mutual information, Blood pressure

## Abstract

**Background:**

Transcriptome-wide association studies (TWASs) have shown great promise in interpreting the findings from genome-wide association studies (GWASs) and exploring the disease mechanisms, by integrating GWAS and eQTL mapping studies. Almost all TWAS methods only focus on one gene at a time, with exception of only two published multiple-gene methods nevertheless failing to account for the inter-dependence as well as the network structure among multiple genes, which may lead to power loss in TWAS analysis as complex disease often owe to multiple genes that interact with each other as a biological network. We therefore developed a Network Regression method in a two-stage TWAS framework (NeRiT) to detect whether a given network is associated with the traits of interest. NeRiT adopts the flexible Bayesian Dirichlet process regression to obtain the gene expression prediction weights in the first stage, uses pointwise mutual information to represent the general between-node correlation in the second stage and can effectively take the network structure among different gene nodes into account.

**Results:**

Comprehensive and realistic simulations indicated NeRiT had calibrated type I error control for testing both the node effect and edge effect, and yields higher power than the existed methods, especially in testing the edge effect. The results were consistent regardless of the GWAS sample size, the gene expression prediction model in the first step of TWAS, the network structure as well as the correlation pattern among different gene nodes. Real data applications through analyzing systolic blood pressure and diastolic blood pressure from UK Biobank showed that NeRiT can simultaneously identify the trait-related nodes as well as the trait-related edges.

**Conclusions:**

NeRiT is a powerful and efficient network regression method in TWAS.

**Supplementary Information:**

The online version contains supplementary material available at 10.1186/s12864-022-08809-w.

## Background

Transcriptome-wide association studies (TWASs) bridge genome-wide association studies (GWASs) and eQTL studies to make inference about the association between the genetically predicted gene expression and the phenotypes [1]. It has shown great promise in interpretation of the GWAS findings and revelation of the underlying mechanisms for disease susceptibility. It is typically done in a two-stage framework where genotype and expression data from an eQTL study are associated as the first stage to obtain the expression prediction weights, followed by the association analysis between the predicted gene expression derived from the weights from the first stage and the outcome GWAS trait. So far many statistical methods have been developed involving both stages, including for the first stage appropriate modeling of SNP effects on gene expression to improve the imputation accuracy (sparse effect as in PrediXcan [2], Bayesian sparse linear model as in TWAS [1], polygenic modeling as in PMR-Egger [3], moPMR-Egger [4], CoMM [5] and nonparametrics as in DPR [6] and TIGAR [7]), constructing a composite instrumental variable [8], leveraging trans-eQTLs [9] or omics mediators [10] and epigenetic annotations [11]; for the second stage using kernel-type method [12, 13], aggregating multiple expression prediction models [14], multiple tissues [15, 16]. In addition, some methods adopted a joint likelihood-based inference procedure to improve the power [3, 4].

Almost all current TWAS methods are univariate in nature with focus on one gene at a time, which may be suboptimal due to its failure to account for the correlation among multiple gene expressions. To our knowledge, there are only two multiple-gene TWAS methods, FOCUS [17] and FOGS [18]. FOCUS extends probabilistic SNP fine-mapping approaches and models the correlation among TWAS signals to obtain risk region-based credible gene sets containing the causal gene at a given confidence level in a Bayesian framework [17]. FOGS conceptually transforms the gene-based fine-mapping into SNPs and performs conditional analysis of each specific *cis*-SNPs in one gene by adjusting the *cis*-SNPs of other genes in the same region [18]. Both FOCUS and FOGS exhibit the great advantage in modeling multiple genes over the TWAS method only modeling one gene at a time. Even so, they are unable to account for the inter-dependence as well as the network structure among multiple genes, thus may leading to loss of power.

A complex disease outcome is seldom the consequence of abnormality involving a single gene but often owing to multiple genes that interact with each other as a biological network whose identification can facilitate better understanding of the pathways in disease etiology. Such a network is conveniently described as a graph in which the nodes and edges are used to represent genes, and physiological interactions between nodes, respectively, so that both the node effects and edge effects can contribute to the diseases [19–22]. It is nontrivial to develop statistical methods in TWAS to detect whether a given biological network is associated with complex disease. One needs to summarize the information underlying the network, to determine a suitable measure to represent the link or connection between two nodes. It should be noted that the link may be nonlinear. We have previously proposed PMINR [22] for efficient network regression analysis, where pointwise mutual information (PMI) is used to measure the strength of the connection between a pair of nodes. PMINR has shown better performance in capturing the general relationship among different nodes in a biological network than other methods including PMNR [22], DGCA [23] and RANK [24]. Specifically, PMNR uses the common linear correlation to represent the between-node connection strength for network regression [22], DGCA is differential gene correlation analysis (i.e., edge effect) to assess the difference in gene regulatory relationships under multiple conditions [23], while RANK can detect the whole pathway due to either correlation or mean changes [24]. However, the modeling framework of PMINR requires all the gene network nodes to be observed, thus cannot be directly implemented for network regression in TWAS analysis, where the gene expression are commonly unobserved in the GWAS.

In this investigation, we developed a Network Regression method in TWAS framework, NeRiT, to detect the association between a given network and phenotypes of interest. It builds upon the two-stage analysis framework that is commonly used in TWAS, first adopts the nonparametric Bayesian Dirichlet process regression (DPR) model in the eQTL study to obtain the SNP effect size estimate on each gene within the network, given that DPR method is robust against the mis-specified distribution of SNP effect size [6]. In addition, we parallelized with Bayesian sparse linear mixed model (BSLMM) model for sensitive analysis [25]. Then, NeRiT adopts PMI to represent the between-node correlation and performs the association analysis with both the node of predicted gene expression and the edge of their correlation among these predicted values to be included in the model. In this case, it can effectively take the network structure into account, and simultaneously identify the trait-related nodes (e.g., genes) as well as the trait-related edges (e.g., gene–gene co-association).

With extensive realistic simulations, we showed that it provides calibrate type I error control for testing either the node effect or the edge effect, yields higher power, especially in testing the edge effect, than the method with product moment representing the between-node correlation. Finally, we applied NeRiT to analyze systolic blood pressure (SBP) and diastolic blood pressure (DBP) from UK Biobank to demonstrate its benefits in real data analysis.

## Results

### Simulations

**Fig. 1:**
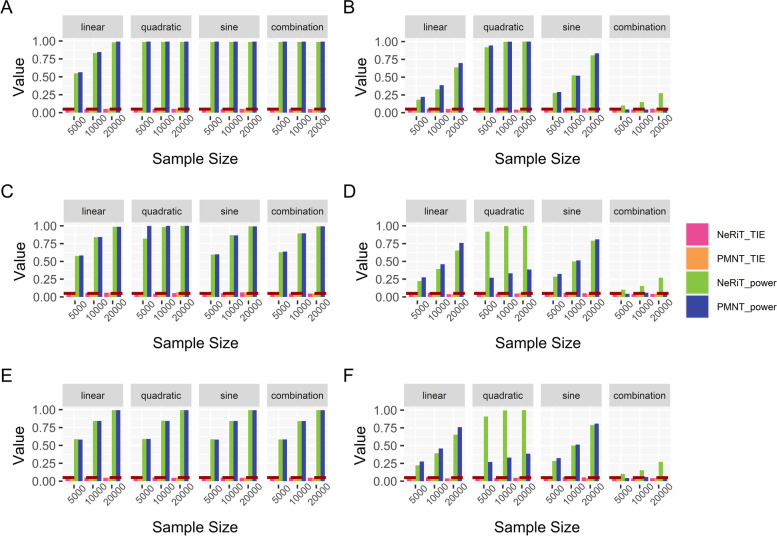
Simulation results of renin secretion network under fixed effecting nodes or edges. Type I error and power of both NeRiT and PMNT with data simulated based on renin secretion network under fixed effecting nodes or edges and four different between-node correlation patterns using DPR as the imputation model in TWAS. The red dotted line represents the significance level ($\mathrm{\alpha }=0.05$). **A** Only node has effect; (**B**) Only edge has effect; the results for effecting node (**C**) or for effecting edge (**D**) when both node and edge change with changing node hanging on the edge; the results for effecting node (**E**) or for effecting edge (**F**) when both node and edge change with changing node not hanging on the edge

Shown in Fig. 1 are the estimated type I error rates and statistical power of NeRiT and PMNT with the data being simulated from renin secretion network and the gene expression prediction model being constructed from DPR model. Here, PMNT is developed by replacing PMI with product moment in the proposed NeRiT framework (i.e. PM-based Network in TWAS, details in Methods). The type I error rates of both two methods were close to the given significance level ($\mathrm{\alpha }=0.05$) under the four simulation scenarios, regardless of the sample size, the linear or nonlinear (quadratic, sine or the combination of quadratic and sine) pattern of correlation. As expected, the power of both two methods increased with sample size under all simulation settings. In addition, both NeRiT and PMNT had comparable power to detect the effecting nodes in the settings when only node has the effect (Fig. 1A) or both node and edge have the effect (Fig. 1C and E). In detecting the effecting edge, the power of NeRiT was a little lower than that of PMNT when the inter-node correlation is linear, which was not surprising as the product moment is the gold standard to describe the inter-node relationship in this case. However, the power of NeRiT was much higher, or at least comparable, than that of PMNT when the inter-node relationship is nonlinear including quadratic, sine, as well as the combination of quadratic and sine (Fig. 1B, D, F). All results were consistent when the effecting node or edges are randomly selected (Figure S1), which illustrated that NeRiT can be reliable and robust against the specific network. In addition, similar conclusions could be drawn when the gene expression prediction model was constructed from BSLMM (Figure S2 and S3).

**Fig. 2:**
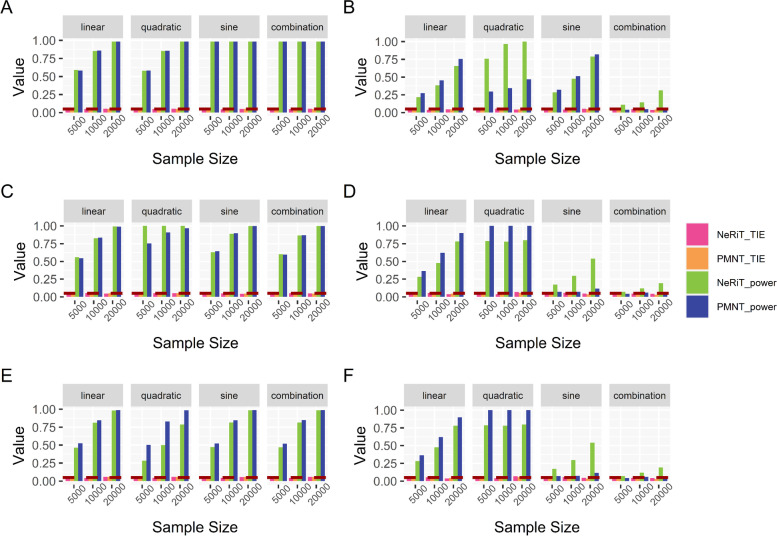
Simulation results of lipid and atherosclerosis network under fixed effecting nodes or edges. Type I error and power of both NeRiT and PMNT with data simulated based on lipid and atherosclerosis network under random effecting nodes or edges and four different between-node correlation patterns using DPR as the imputation model in TWAS. The red dotted line represents the significance level ($\mathrm{\alpha }=0.05$). **A** Only node has effect; (**B**) Only edge has effect; the results for effecting node (**C**) or for effecting edge (**D**) when both node and edge change with changing node hanging on the edge; the results for effect node (**E**) or for effecting edge (**F**) when both node and edge change with changing node not hanging on the edge

Such findings were also made when the data are simulated from lipid and atherosclerosis network (Fig. 2). In addition, all the results were consistent either when the effecting node or edges are randomly selected (Figure S4) or when the gene expression prediction model was constructed from BSLMM (Figure S5 and S6). Therefore, all the simulation results illustrated that NeRiT was robust against both the network size and network structure.

### Applications

Shown in Table 1 are the results of the network regression in detecting the association between renin secretion network and the blood pressure traits in TWAS framework by integrating GEUVADIS data and UK Biobank GWAS. Consistent with simulations, both NeRiT and PMNT successfully detected the same node genes at a significance level of 0.05, including *CREB1* ($p=0.001\mathrm{\;and \;}0.002$ for NeRiT and PMNT, respectively) and *ADRB1* ($p=0.029\mathrm{\; and \;}0.025$ for NeRiT and PMNT, respectively) for SBP and *ADRB1* ($p=2.540\times {10}^{-5}\mathrm{ and\; }2.565\times {10}^{-5}$ for NeRiT and PMNT, respectively) for DBP. For SBP, PMNT successfully identified an effecting edge *GNAS-ADCY5* ($p=0.025$), which, to a lesser extent, has also been detected by NeRiT ($p=0.055$). For DBP, PMNT failed to detect any effecting edges, while NeRiT successfully identify the effecting edge *GNAS-ADCY5* ($p=0.024$) and, to a lesser extent, *GNAS-PTGER2* ($p=0.049$). The scatter plots describing the relationship between the expression of GNAS and ADCY5 in eQTL study, as well as GNAS and PTGER2, are displayed as supplementary Figure S7 and S8, which indicates that there is no linear relationship between these genes. This highlights the important feature of PMI in capturing the nonlinear relationship and the power advantage of NeRiT.

**Table 1:** Renin secretion network regression of both methods with *p* values in parenthesis

Trait		NeRiT	PMNT
**SBP**	**Nodes**	*CREB1* ($0.001$) *ADRB1* ($0.029$)	*CREB1* ($0.002$) *ADRB1* ($0.025$)
	**Edges**	*GNAS-ADCY5* ($0.055$)	*GNAS-ADCY5* ($0.025$)
**DBP**	**Nodes**	*ADRB1* ($2.540\times {10}^{-5}$)	*ADRB1* ($2.565\times {10}^{-5}$)
	**Edges**	*GNAS-ADCY5* ($0.024$) *GNAS-PTGER2* ($0.049$)	

Shown in Table 2 are the results of the network regression in detecting the association between the aldosterone-regulated sodium reabsorption network and the blood pressure traits. Consistent with simulations, both NeRiT and PMNT successfully identified the same genes at a significance level of 0.05, including *IGF1* ($p=0.020\mathrm{ \;and\; }0.021$ for NeRiT and PMNT, respectively), *MAPK1* ($p=0.028\mathrm{\; and \;}0.027$ for NeRiT and PMNT, respectively), SLC9A3R2 ($p=0.039\mathrm{\; and\; }0.040$ for NeRiT and PMNT, respectively) and *IRS1* ($p=0.047\mathrm{\; and\; }0.048$ for NeRiT and PMNT, respectively) for SBP, and *NEDD4L* ($p=0.013$ for both NeRiT and PMNT) for DBP. For DBP, NeRiT successfully identified the effecting edge *SGK1-NR3C2* ($p=0.044$), while PMNT failed to detect any effecting edges. Again, Figure S9 shows the scatter plot of gene expression relationship between *SGK1* and *NR3C2* in eQTL study, which also shows the inter-node correlation is nonlinear. Again, all results were similar when using BSLMM as the gene expression prediction model (Table S1 and Table S2).

**Table 2:** Aldosterone-regulated sodium reabsorption network regression of both methods with *p* values in parenthesis

Trait		NeRiT	PMNT
**SBP**	**Nodes**	*IGF1* ($0.020$) *MAPK1* ($0.028$) *SLC9A3R2* ($0.039$) *IRS1* ($0.047$)	*IGF1* ($0.021$) *MAPK1* ($0.027$) *SLC9A3R2* ($0.040$) *IRS1* ($0.048$)
	**Edges**		
**DBP**	**Nodes**	*NEDD4L* ($0.013$)	*NEDD4L* ($0.013$)
	**Edges**	*SGK1-NR3C2* ($0.044$)	

## Discussion

We have presented NeRiT, a novel network regression method that detects the association between a given network and the phenotypes of interest in TWAS. It is a key step in TWAS analysis to choose the appropriate prior distribution of genotype effect size to predict gene expression, and it is often hard to determine the appropriate prior distribution of the genotype effect size since the real genetic structure is scarcely known. For network regression in TWAS, NeRiT relies on DPR to obtain the gene expression prediction weights with PMI to measure the between-node correlation and can simultaneously identify the specific gene nodes as well as edges related to the outcome traits. Comprehensive simulations illustrated that PMI can capture the general relationship among different gene nodes, and NeRiT has better performance than other competing methods.

One may be tempting to first get the PMI estimates among the network nodes of gene expression in the eQTL study, rather than among the network nodes of predicted gene expression in GWAS, given that the gene expression data are available in the eQTL study. Then, the estimate of PMI can be considered as a new exposure and the standard TWAS analysis can be conducted. However, there would be large prediction error due to the limited sample size in the eQTL study (e.g. only 465 samples in the GEUVADIS data). In addition, different from traditional TWAS analysis naturally choosing the cis-SNPs of each gene as the genotypes, it is hard to determine, both biologically and statistically, which SNPs can be chosen for the PMI between two genes as the genotypes.

Findings in our real data analysis were consistent with previous work. Loss of CREB content and function is a common, pathogenic vascular smooth muscle cells response to cardiovascular risk factors [26]. Hypertension is a multifactorial disease with a substantial genetic component. *ADRB1* is important in the regulation of blood pressure, cardiovascular function and lipid metabolism [27], and it was found that individuals with higher expression of the *ADRB1* receptor gene are at increased risk of hypertension [28]. *GNAS* implicated in variable blood pressure lowering of drug therapy in cardiovascular medicine [29]. Previous studies indicated that targeted disruption of *PTGER2* results in hypertension [30]. GNAS-ADCY5 plays a key role in a wide variety of inter-connected pathways including PKA signaling and cAMP signaling, which have well-established roles in the control of blood pressure [31].

*IGF1* implicated in essential hypertension [29]. *MAPK1* stimulates cardiac fibroblast and myofibroblast growth, thus contributing to the pathological actions of aldosterone in the myocardium [32]. *SLC9A3R2* is associated with SBP and/or DBP and with consistent directions of effect for SBP and DBP [33]. *NEDD4L* controls blood pressure by downregulating renal epithelial sodium channel (ENaC) expression and inhibiting sodium reabsorption, and some genetic variations in *NEDD4L* could influence the ability of the NEDD4L protein, which is significantly associated with an increased risk for adverse cardiovascular outcomes [34]. Moreover, *SGK1* phosphorylates and inactivates the ubiquitin ligase NEDD4L to reduce its interaction with the epithelial sodium channel. This consequently increases cell surface expression of the *ENaC* and thus sodium reabsorption across the apical membrane, enabling regulation of blood pressure in response to aldosterone [35].

NeRiT is not without limitations. First, the gene network structure is assumed to be known. In fact, learning gene network structure requires determining every possible edge with the highest degree of data matching, and a joint probability distribution of gene network nodes can reflect more than one network structure. Indeed, most biologists can roughly describe the specific network for the corresponding biological process, and publicly available multiple databases (such as KEGG) can also be helpful to establish the network structure. Second, the inference of PMINR directly plugs the estimate of correlation among different predicted gene expression into the regression model and fails to account for the uncertainty during such correlation estimate, such inference procedure may lead to the biased estimate and power loss, especially in smaller sample size. Meanwhile, it ignores the direction of the link between gene codes. Third, we adjusted the *p* values in the real data application using Bonferroni correction as well as the FDR, but almost no significant node or edge can be detected (Tables S3, S4, S5, S6, S7, S8, S9 and S10). For network regression in TWAS, the node test and the edge test are often highly correlated, with further exacerbation since the gene expression are predicted using the cis-SNP of each gene, which are often in linkage disequilibrium. It is not straightforward to correct the *p* value or control the FDR. It is desirable to develop methods that can calculate the effective number of independent tests, to further address the multiple testing issue. In addition, caution should be made against the interpretation of the effect of individual node and edge, given the potential for mediation effects within the network.

## Conclusions

In conclusion, NeRiT is a powerful and efficient network regression method in TWAS.

## Methods

### An overview

NeRiT concerns about network regression analysis in TWAS to identify the trait-associated biological network involving multiple genes from a network medicine perspective. Specifically, assume that we have a biological network with $m$ nodes (the magnitude of each gene’s expression in the regulation network) and $l$ edges (the strength of between-node connection). We denote ${{\varvec{X}}}_{i}\left(i=\mathrm{1,2},\dots ,m\right)$ as an ${n}_{1}$-vector of gene expression measurements for the $i$-th gene, that is measured on ${n}_{1}$ individuals in the gene expression study and denote ${{\varvec{g}}}_{i}$ as an ${n}_{1}$ by ${p}_{i}$ matrix of genotypes for ${p}_{i}$ *cis*-SNPs of the $i$-th gene in the same study; ${{\varvec{\eta}}}_{i}$ is a ${p}_{i}$-vector of SNP effect sizes on the ${{\varvec{X}}}_{i}$. We assume ${{\varvec{X}}}_{i}$ has been standardized with a mean of zero and a variance of one. We denote ${\varvec{y}}={\left({y}_{1},{y}_{2},\dots ,{y}_{{n}_{2}}\right)}^{{\varvec{T}}}$ as an ${n}_{2}$-vector of outcome variable (i.e. trait) that is measured on ${n}_{2}$ individuals in the GWAS and denote ${{\varvec{G}}}_{i}$ as an ${n}_{2}$ by ${p}_{i}$ matrix of genotypes for the same ${p}_{i}$ SNPs of the $i$-th gene. ${{\varvec{G}}}_{ki} \left(k=\mathrm{1,2},\dots ,{n}_{2}; i=\mathrm{1,2},\dots ,m\right)$ denotes a $1$ by ${p}_{\mathrm{i}}$ matrix of genotypes of the $i$-th gene for the $k$-th individual; ${Z}_{ks}\boldsymbol{ }(s=\mathrm{1,2},\dots , S)$ denotes the $s$-th covariate for the $k$-th individual; ${E}_{kij}$ denotes the estimator of PMI between node ${X}_{i}$ and node ${X}_{j}$ for the $k$-th individual (details in below).

NeRiT considers two linear regressions to model the gene expression study and GWAS separately in TWAS,

1 $${{\varvec{X}}}_{i}={{\varvec{g}}}_{i}{{\varvec{\eta}}}_{i}+{{\varvec{\varepsilon}}}_{{{\varvec{X}}}_{i}},\boldsymbol{ }\left(i=\mathrm{1,2},\dots ,m\right)$$

for subject $$k \left(k=\mathrm{1,2},\dots ,{n}_{2}\right)$$,

2 $${y}_{k}={\beta }_{0}+\sum_{s=1}^{S}{Z}_{ks}{\alpha }_{ks}+\sum_{i=1}^{m}{{\varvec{G}}}_{ki}{\widehat{{\varvec{\eta}}}}_{i}{\beta }_{i}+\sum_{i=1}^{m}\sum_{j>i}^{m}{I}_{ij}{E}_{kij}{\gamma }_{ij}+{e}_{k}$$

where

3 $${I}_{ij}=\left\{\begin{array}{cc}1& {{\varvec{X}}}_{i}\text{ and }{{\varvec{X}}}_{j} \, \text{are connected in the network }\\ 0& \text{ otherwise}\end{array}\right.$$

Equations (1) and (2) are for the gene expression data and the GWAS data, respectively. Here, ${\beta }_{0}$ is a constant of intercept,${\alpha }_{ks}$ is the coefficients of the $s$-th covariates for the $k$-th individual; ${\widehat{{\varvec{\eta}}}}_{i}$ is the estimate of the SNP effect size on the $i$-th gene estimated by the prediction model; ${\beta }_{i}$ is the causal effect of the $i$-th gene; ${\gamma }_{ij}$ is the effect between the $i$-th gene and $j$-th gene. ${{\varvec{\varepsilon}}}_{{{\varvec{X}}}_{i}}$ is an ${n}_{1}$-vector of residual error with each element independently and identically distributed from a normal distribution$N\left(0,{\sigma }_{X}^{2}\right)$; $e=({e}_{1},{e}_{2},\dots ,{e}_{{n}_{2}})$ is an ${n}_{2}$-vector of residual error with each element independently and identically distributed from a normal distribution$N\left(0,{\sigma }_{y}^{2}\right)$. Since gene expression data is unobserved in GWAS, we denote ${\widehat{{\varvec{X}}}}_{i}={\widehat{{\varvec{\eta}}}}_{i}{\beta }_{i}$ as an ${n}_{2}$-vector of predicted gene expressions for the $i$-th gene, where the SNP effect ${\widehat{{\varvec{\eta}}}}_{i}$ needs to be obtained by the gene expression prediction model (details regarding the gene expression prediction model are provided below). A key feature of NeRiT is the integration of using gene expression prediction model in the first stage and using PMI in the second stage for network regression in TWAS. It should be noted that NeRiT decomposes the change of the whole biological network into the gene node and edge changes in TWAS framework, and naturally incorporated the network structure into the model. In addition, Wald test was used to identify the gene nodes or edges that are related to the outcome traits. The NeRiT is implemented in the R package NeRiT, freely available on GitHub (https://github.com/XiuyuanJin/NeRiT).

### Gene expression prediction model in TWAS

As accuracy of prediction model of gene expression is quite important for the performance of TWAS, improvement in the prediction can substantially increase the power of TWAS [36]. Different prediction models essentially differ in their assumptions about the prior distribution of the SNP effect size. In theory, the accuracy of prediction model depends on how close the prior distribution is to the real genetic structure, which is often unknown. Indeed, there are many differences in heritability, minor allele frequency and effect size across different complex traits or diseases. Therefore, most of the existing parametric models (e.g., linear mixed model), which often use a prior effect size distribution represented by several parameters, are not sufficient to capture the true distribution of SNP effect size underlying the genetic data. In this study, we chose the non-parametric Dirichlet process regression (DPR), to construct gene expression prediction models.

DPR relies on the Dirichlet process to flexibly model the effect size distribution using infinitely many parameters and is therefore able to infer the effect size distribution from the data at hand. In addition, to investigate whether the performance of NeRiT can be influenced by the gene expression models, we alternatively adopt the commonly used Bayesian sparse linear mixed model (BSLMM), which assumes the SNP effect size on gene expressions follows two mixture normal distributions, to construct the gene expression prediction model.

### Pointwise mutual information with the kernel density estimator

PMI has been illustrated to have better performance than other metrics in capturing the general relationship (linear or nonlinear) among different nodes in a biological network. For any two random variables $X$ and $Y$, PMI is defined as follows [37]:

4 $$PMI\left(x;y\right)=\mathrm{log}\frac{p\left(x;y\right)}{p\left(x\right)p\left(y\right)}$$

where $p\left(x;y\right)$ is the joint distribution of $\mathrm{X}$ and $\mathrm{Y}$, $p\left(x\right)$ and $p\left(y\right)$ are the marginal distributions of $\mathrm{X}$ and $\mathrm{Y}$, respectively. Statistically, PMI can extract the general non-independency of two variables. We need to estimate the two-dimensional joint density function and marginal density function for a given sample to calculate the PMI between two network nodes. To guard against the misspecification of distribution, we chose the non-parametric kernel density estimation to characterize the corresponding distribution based on the data at hand and to improve the robustness of the PMI estimator.

The two-dimensional kernel density estimation is defined as

5 $${\widehat{f}}_{H}({\varvec{x}})=\frac{1}{{n}_{2}}\sum_{k=1}^{{n}_{2}}{K}_{H}\left({{\varvec{X}}}_{k}-{\varvec{x}}\right)$$

where ${{\varvec{X}}}_{k}=({X}_{ki},{X}_{kj}), k=\mathrm{1,2},...,{n}_{2}, i\ne j, i,j=\mathrm{1,2},...,m$ is the $k$-th sample of the $i$-th and $j$-th node, respective; $H$ is a 2 by 2 bandwidth matrix, which is symmetric and positive definite; $K$ is a bivariate kernel function and ${K}_{H}\left({\varvec{x}}\right)={\left|H\right|}^{-\frac{1}{2}}K\left({H}^{-\frac{1}{2}}{\varvec{x}}\right)$. Here we chose the commonly-used two-dimensional normal kernel as follows:

6 $${K}_{H}\left({\varvec{x}}\right)={\left(2\pi \right)}^{-\frac{d}{2}}{\left|H\right|}^{-\frac{1}{2}}\mathrm{exp}\left(-\frac{1}{2}{{\varvec{x}}}^{T}{H}^{-1}{\varvec{x}}\right)$$

### Simulation

**Fig. 3:**
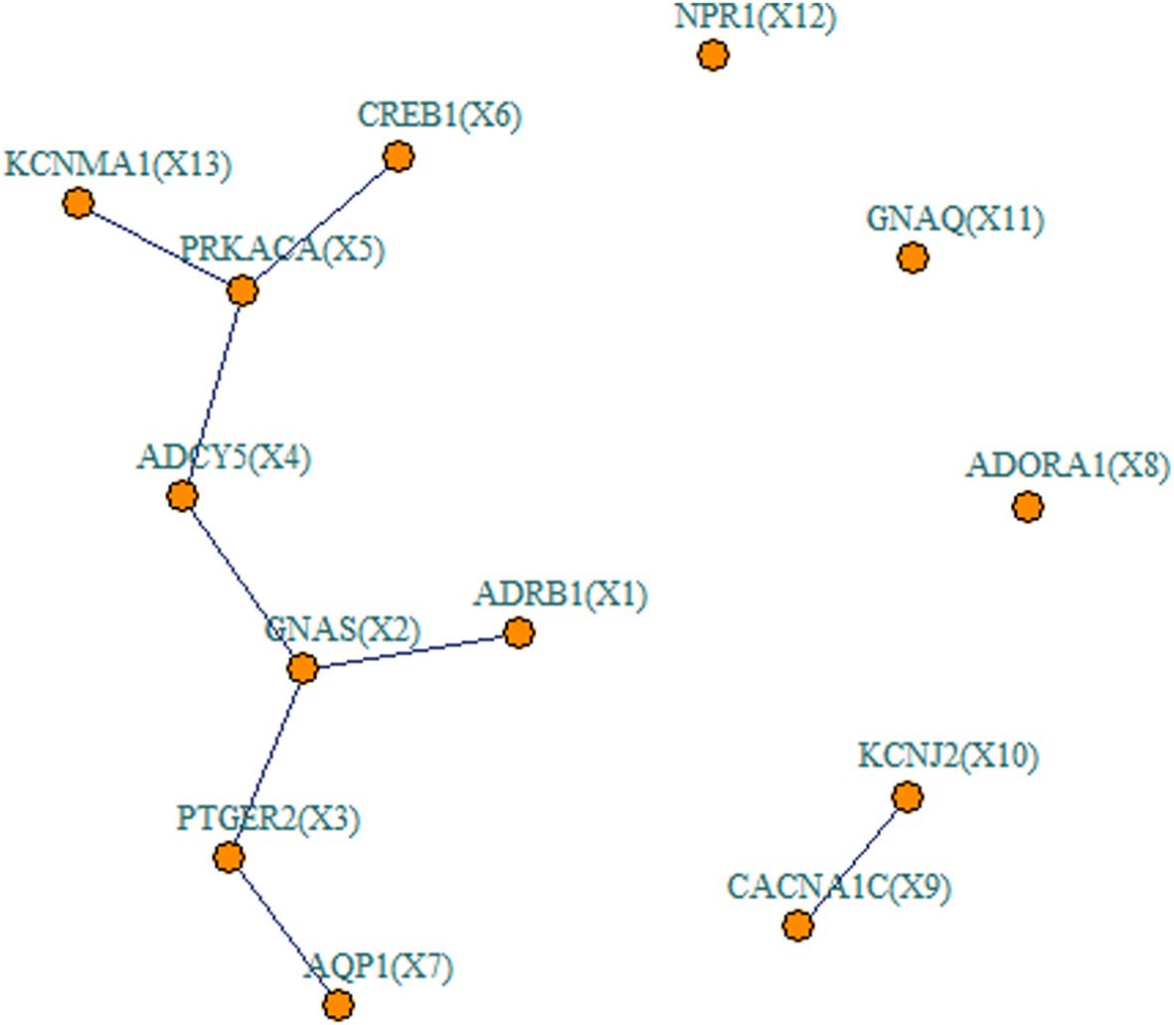
The network structure based on the renin secretion pathway from KEGG

**Fig. 4:**
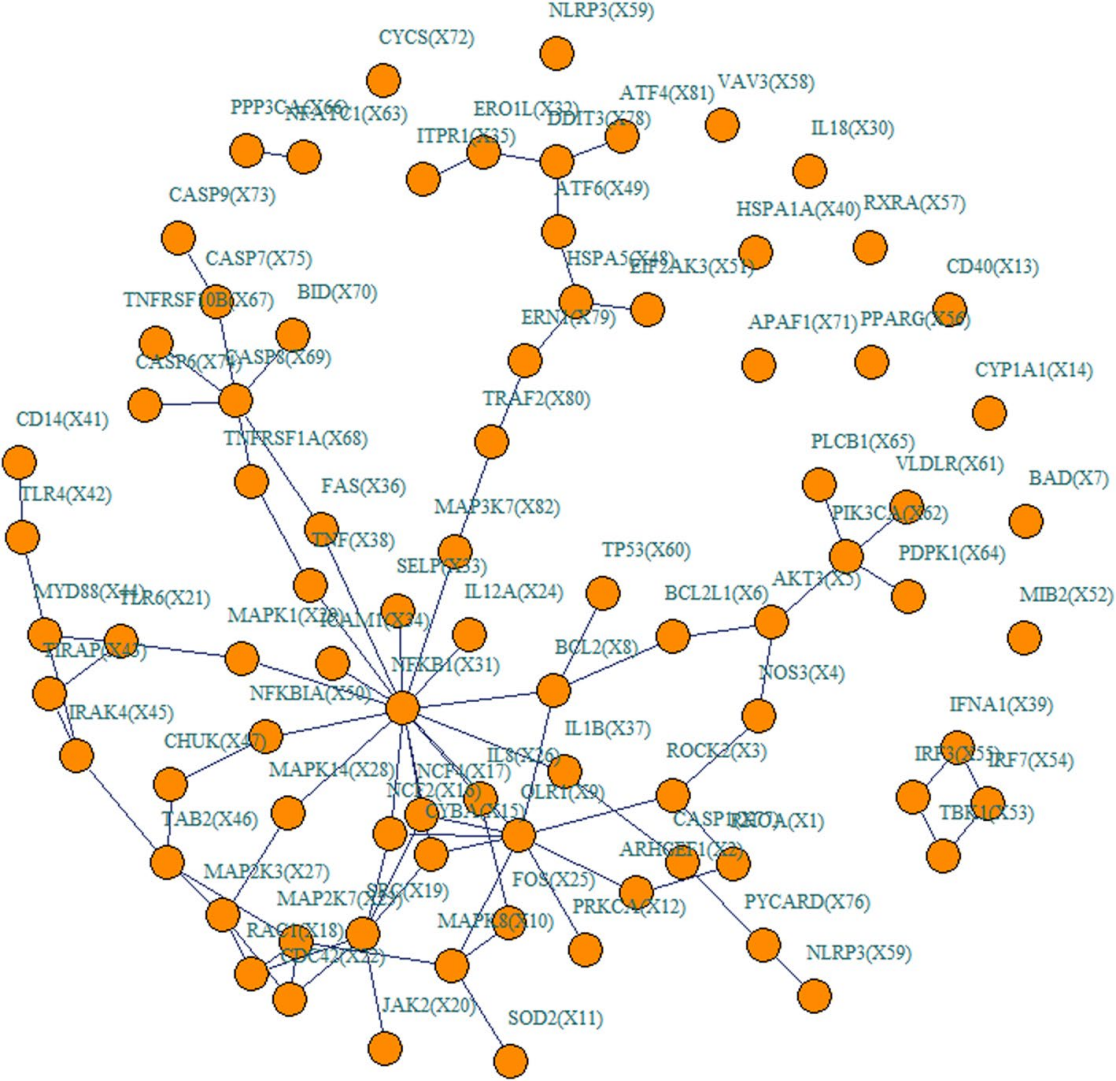
The network structure based on the lipid and atherosclerosis pathway from KEGG

Given that there are no statistical methods for network TWAS analysis yet, we performed comprehensive simulations to compare the performance of NeRiT with the method that replaces PMI with product moment in NeRiT framework (term as PM-based Network in TWAS (PMNT)). We chose this method for comparison as product moment (i.e. Pearson correlation) is commonly used to describe the dependence between two network nodes [38–41]. To make our simulation more realistic, we first mimicked a TWAS analysis by integrating the GEUVADIS [42] data with GWAS from UK Biobank [43] (details regarding these two datasets are provided below). We obtained genotype data and gene expression data from GEUVADIS and standardized the genotype and expression vector of each SNP to have a zero mean and a unit standard deviation. We then applied DPR or BSLMM to obtain the estimate of the SNP effect size ${\widehat{{\varvec{\eta}}}}_{i}$ on gene expression, respectively. Then, we obtained genotypes for the same SNPs from UK Biobank and standardized the genotype vector of each SNP to have a zero mean and a unit standard deviation. With the standardized genotype matrix and weights vectors ${\widehat{{\varvec{\eta}}}}_{i}$ from the previous step, we obtained the predicted gene expression. In addition, to avoid the risk of pre-specifying the network structure, we selected a realistic small network of renin secretion (Entry: hsa04924) with 13 gene nodes and 8 edges (Fig. 3) and a large network of lipid and atherosclerosis (Entry: hsa05417) with 82 gene nodes and 87 edges (Fig. 4) from Kyoto Encyclopedia of Genes and Genomes (KEGG, http://www.kegg.jp/kegg/kegg1.html), respectively. Note that we overlapped these network genes with those in the above mimicking TWAS framework to re-formulate the two biological networks.

We considered the following four scenarios for simulation:

- (1) only nodes of network having the effect (e.g., node ${X}_{1}$ in Fig. 3),
- (2) only edges of network having the effect (e.g., edge ${E}_{\mathrm{2,4}}$ in Fig. 3),
- (3) both nodes and edges of network having effect, with the nodes hanging on the edge (e.g., node ${X}_{13}$ and edge ${E}_{\mathrm{5,13}}$ in Fig. 3),
- (4) both nodes and edges of network having effect, with the nodes not hanging on the edge (e.g., node ${X}_{12}$ and edge ${E}_{\mathrm{5,13}}$ in Fig. 3).

In each scenario, we use four inter-node relationship patterns, including the linear correlation, the quadratic relationship (${X}_{j}=0.5\cdot {{X}_{i}}^{2}+\varepsilon$), the sine relationship (${X}_{j}=\mathrm{sin}{X}_{i}+\varepsilon$) as well as the combination of quadratic and sine relationship (${X}_{j}={(\mathrm{sin}{X}_{i})}^{2}+\varepsilon$), where $\varepsilon$ is the residual error from a standard normal distribution $\varepsilon \sim N(\mathrm{0,1})$. For example, if we assign the quadratic relationship between node ${X}_{5}$ and node ${X}_{13}$, then ${X}_{13}=0.5\cdot{{X}_{5}}^{2}+\varepsilon$. The nonlinear quadratic relationship between ${X}_{5}$ and ${X}_{13}$ can be transformed to the linear relationship between ${{X}_{5}}^{2}$ and ${X}_{13}$, we then set ${E}_{\mathrm{5,13}}=0.5\cdot {{X}_{5}}^{2}\cdot {X}_{13}$ to represent the edge variable ${E}_{\mathrm{5,13}}$ to simulate the traits. The type I error rate was assessed under the null hypothesis, with all node and edge effects set to be $0$($\beta =0$, $\gamma =0$), followed by the assessment of power with $\beta =0.03$, $\gamma =0.03$.

We performed 1000 simulation replicates under different sample sizes (5000, 10,000, 20,000) for each simulation replicate above. Besides pre-specifying the effecting nodes and edges, we further consider additional cases under the same above settings but randomly select the effecting nodes or edges, to eliminate the impact of network structures.

### Application

We applied NeRiT through integrating gene expression data from GEUVADIS with GWASs from UK Biobank. Specifically, we obtained the GEUVADIS data as the gene expression data and examined two traits from the UK Biobank. The detailed data processing steps for the GEUVADIS data and UK Biobank data are described below.

The GEUVADIS data contains gene expression measurements for 465 individuals collected from five different populations that include CEPH (CEU), Finns (FIN), British (GBR), Toscani (TSI), and Yoruba (YRI). It performed mRNA and small RNA sequencing on 465 Epstein-Barr-virus-transformed lymphoblastoid cell line samples from five populations, and the genotype data was from the 1000 Genomes project. In the expression data, we only focused on protein coding genes and lncRNAs that are annotated in GENCODE (release 12) [44, 45]. Among these genes, we removed low-expressed genes that have zero counts in at least half of the individuals to obtain a final set of 15,810. We, following Zeng and Zhou [6], first quantile normalized the gene expression across individuals in each population to a standard normal distribution, and then normalized the gene expression to a standard normal distribution across individuals from five populations. To further remove the technical variations and batch effects, we performed PEER normalization to remove latent confounding factors for samples from five populations since the original gene expression measurements were read counts.

Besides the expression data, all individuals in GEUVADIS also have their genotypes sequenced in the 1000 Genomes Projects. We obtained genotype data from the 1000 Genomes Project phase 3. We filtered out SNPs that have a Hardy–Weinberg equilibrium (HWE) $p$ value $<{10}^{-4}$, a genotype call rate $<95\mathrm{\%}$, or a minor allele frequency (MAF) $<0.001$. We retained a total of 7,072,917 SNPs for analysis.

The UK Biobank data consists of 487,298 individuals and 92,693,895 imputed SNPs [43]. We followed the same sample QC procedure in Neale lab (Web Resources) to retain a total of 337,129 individuals of European ancestry. We filtered out SNPs with an HWE $p$ value $<$ ${10}^{-7}$, a genotype call rate $<$ $95\%$, or an MAF $<$ $0.001$ to obtain a total of 13,876,958 SNPs. For each trait in turn, we regressed the resulting standardized phenotypes on sex and top genotype principal components (PCs) to obtain the residuals, standardized the residuals to have a mean of zero and a standard deviation of one, and finally used these scaled residuals to conduct TWAS analysis.

We integrated the GEUVADIS data with GWAS from UK Biobank for TWAS analysis. For each gene in turn in the GEUVADIS data, we extracted *cis*-SNPs that are within either 100 kb upstream of the transcription start site (TSS) or 100 kb downstream of the transcription end site (TES). We overlapped these *cis*-SNPs of genes in GEUVADIS with the SNPs obtained from UK Biobank to obtain common sets of SNPs.

**Fig. 5:**
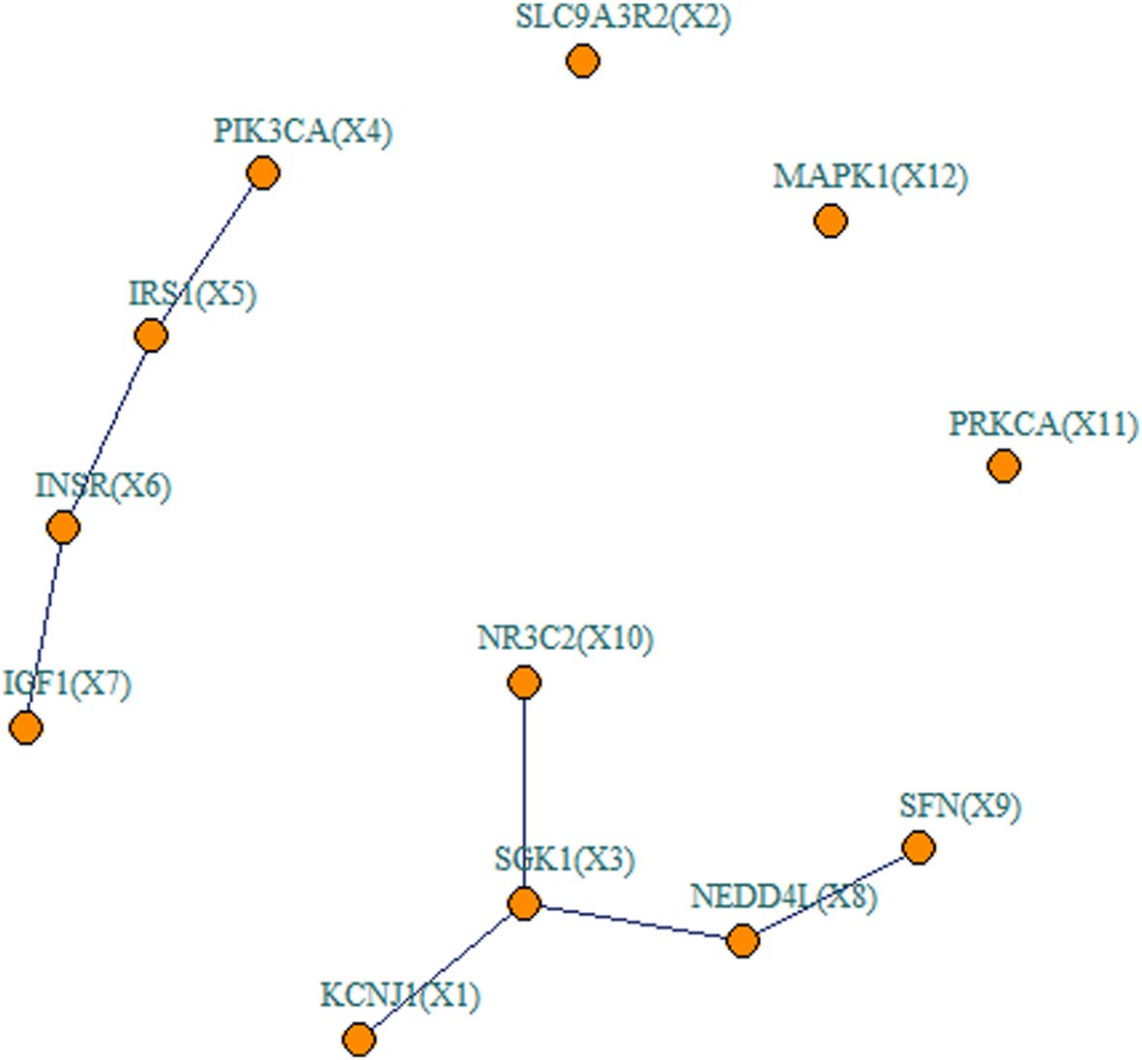
The network structure based on the aldosterone-regulated sodium reabsorption pathway from KEGG

Here we focused on the UK Biobank GWAS of systolic blood pressure (SBP) and diastolic blood pressure (DBP) to investigate the association between the blood pressure and two biological networks from KEGG, one is renin secretion network (Entry: hsa04924) with 13 gene nodes and 8 edges (Fig. 3), the other is aldosterone-regulated sodium reabsorption (Entry: hsa04960) network with 12 gene nodes and 7 edges (Fig. 5). Note that we overlapped these network genes with those in the above mimicking TWAS framework to re-formulate the two biological networks. Cardiovascular diseases are a leading cause of death globally. The reason that we chose the blood pressure traits is that elevated blood pressure is a major risk factor for cardiovascular morbidity and mortality [46]. The SNP heritability of blood pressure was estimated in the range of 0.3–0.5 in previous studies [47]. In addition, the renin–angiotensin–aldosterone system (RAAS) is a critical regulator of blood volume and systemic vascular resistance. RASS is composed of renin, angiotensin and aldosterone, these three major compounds act together to elevate arterial pressure in response to decreased renal blood pressure, decreased salt delivery to the distal convoluted tubule, and/or beta-agonism. Through these mechanisms, the body can elevate blood pressure in a prolonged manner [48]. It should be noted that one important feature of NeRiT was that NeRiT can detect whether the whole network or gene or inter-gene correlation is associated with the blood pressure traits.

### Web Resources

KEGG, www.kegg.jp/kegg/kegg1.html

GEUVADIS, http://www.geuvadis.org

UK Biobank, https://www.ukbiobank.ac.uk/

Sample QC procedure in Neale lab, https://github.com/Nealelab/UK_Biobank_GWAS/tree/master/imputed-v2-gwas

## Supplementary Information

**Additional file 1: Figure S1.** Simulation results of renin secretion network under random effecting nodes or edges. **Figure S2.** Simulation results of renin secretion network under fixed effecting nodes or edges. **Figure S3**. Simulation results of renin secretion network under random effecting nodes or edges. **Figure S4**. Simulation results of lipid and atherosclerosis network under random effecting nodes or edges. **Figure S5**. Simulation results of lipid and atherosclerosis network under fixed effecting nodes or edges. **Figure S6**. Simulation results of lipid and atherosclerosis network under random effecting nodes or edges. **Figure S7.** The scatter plots of relationship between the expression of *GNAS* and *ADCY5* in eQTL study. **Figure S8.** The scatter plots of relationship between the expression of *GNAS* and *PTGER2* in eQTL study. **Figure S9.** The scatter plots of relationship between the expression of *SGK1* and *NR3C2* in eQTL study. **Table S1.** Renin secretion network regression of both methods with *p *values in parenthesis. **Table S2.** Aldosterone-regulated sodium reabsorption network regression of both methods with *p *values in parenthesis. **Table S3.** Results of the renin secretion network regression on SBP using DPR as the imputation model. **Table S4.** Results of the renin secretion network regression on DBP using DPR as the imputation model. **Table S5.** Results of the renin secretion network regression on SBP using BSLMM as the imputation model. **Table S6.** Results of the renin secretion network regression on DBP using BSLMM as the imputation model. **Table S7.** Results of the aldosterone-regulated sodium reabsorption network regression on SBP using DPR as the imputation model. **Table S8.** Results of the aldosterone-regulated sodium reabsorption network regression on DBP using DPR as the imputation model. **Table S9.** Results of the aldosterone-regulated sodium reabsorption network regression on SBP using BSLMM as the imputation model. **Table S10.** Results of the aldosterone-regulated sodium reabsorption network regression on DBP using BSLMM as the imputation model.

## Data Availability

No data were generated in the present study. The GEUVADIS gene expression data are publicly available online. The UK Biobank data is from UK Biobank resource under application number 51470.

## Abbreviations

TWAS: Transcriptome-wide association study
GWAS: Genome-wide association study
eQTL: Expression quantitative trait locus
PMI: Pointwise mutual information
DPR: Dirichlet process regression
BSLMM: Bayesian sparse linear mixed model
SBP: Systolic blood pressure
DBP: Diastolic blood pressure
KEGG: Kyoto Encyclopedia of Genes and Genomes
SNP: Single nucleotide polypeptide
RAAS: Renin–angiotensin–aldosterone system
