# Correction: Network regression analysis in transcriptome-wide association studies

**DOI:** 10.1186/s12864-022-08844-7

**Published:** 2022-08-25

**Authors:** Xiuyuan Jin, Liye Zhang, Jiadong Ji, Tao Ju, Jinghua Zhao, Zhongshang Yuan

**Affiliations:** 1 Department of Biostatistics, School of Public Health, Cheeloo College of Medicine, Shandong University, Jinan, 250012 Shandong China; 2 Institute for Medical Dataology, Shandong University, Jinan, 250003 Shandong China; 3 Institute for Financial Studies, Shandong University, Jinan, 250100 Shandong China; 4 Department of Public Health and Primary Care, Cardiovascular Epidemiology Unit, University of Cambridge, Cambridge, UK

**Correction: BMC Genomics 23, 562 (2022)**

**https://doi.org/10.1186/s12864-022-08809-w**

Following publication of the original article [1], it was reported that the PDF versions of Fig. 1 and Fig. 2 were incorrect and therefore did not match the correct, HTML versions due to a typesetting error.

The PDF version of the original article [1] has been updated.
