# Supplementary material for: Network regression analysis in transcriptome-wide association studies
Source: BMC Genomics. 2022 Aug 6;23:562. doi: 10.1186/s12864-022-08809-w (PMC9356418; doi:10.1186/s12864-022-08809-w)
Supplement: Supplementary file 1 — Additional file 1: Figure S1. Simulation results of renin secretion network under random effecting nodes or edges. Figure S2. Simulation results of renin secretion network under fixed effecting nodes or edges. Figure S3. Simulation results of renin secretion network under random effecting nodes or edges. Figure S4. Simulation results of lipid and atherosclerosis network under random effecting nodes or edges. Figure S5. Simulation results of lipid and atherosclerosis network under fixed effecting nodes or edges. Figure S6. Simulation results of lipid and atherosclerosis network under random effecting nodes or edges. Figure S7. The scatter plots of relationship between the expression of GNAS and ADCY5 in eQTL study. Figure S8. The scatter plots of relationship between the expression of GNAS and PTGER2 in eQTL study. Figure S9. The scatter plots of relationship between the expression of SGK1 and NR3C2 in eQTL study. Table S1. Renin secretion network regression of both methods with p values in parenthesis. Table S2. Aldosterone-regulated sodium reabsorption network regression of both methods with p values in parenthesis. Table S3. Results of the renin secretion network regression on SBP using DPR as the imputation model. Table S4. Results of the renin secretion network regression on DBP using DPR as the imputation model. Table S5. Results of the renin secretion network regression on SBP using BSLMM as the imputation model. Table S6. Results of the renin secretion network regression on DBP using BSLMM as the imputation model. Table S7. Results of the aldosterone-regulated sodium reabsorption network regression on SBP using DPR as the imputation model. Table S8. Results of the aldosterone-regulated sodium reabsorption network regression on DBP using DPR as the imputation model. Table S9. Results of the aldosterone-regulated sodium reabsorption network regression on SBP using BSLMM as the imputation model. Table S10. Results of the aldosterone-regulated sodium reabsorption [file 12864_2022_8809_MOESM1_ESM.docx]

**Supplemental Figures**

**

**

**Figure S1 | Simulation results of renin secretion network under random effecting nodes or edges.** Type I error and power of both NeRiT and PMNT with data simulated based on renin secretion network under random effecting nodes or edges and four different between-node correlation patterns using DPR as the imputation model in TWAS . The red dotted line represents the significance level ($\alpha=0.05$). (A) Only node has effect; (B) Only edge has effect; the results for effecting node (C) or for effecting edge (D) when both node and edge change with changing node hanging on the edge; the results for effecting node (E) or for effecting edge (F) when both node and edge change with changing node not hanging on the edge.





**Figure S2 | Simulation results of renin secretion network under fixed effecting nodes or edges.** Type I error and power of both NeRiT and PMNT with data simulated based on renin secretion network under fixed effecting nodes or edges and four different between-node correlation patterns using BSLMM as the imputation model in TWAS. The red dotted line represents the significance level ($\alpha=0.05$). (A) Only node has effect; (B) Only edge has effect; the results for effecting node (C) or for effecting edge (D) when both node and edge change with changing node hanging on the edge; the results for effecting node (E) or for effecting edge (F) when both node and edge change with changing node not hanging on the edge.

**

Figure S3 | Simulation results of renin secretion network under random effecting nodes or edges.** Type I error and power of both NeRiT and PMNT with data simulated based on renin secretion network under random effecting nodes or edges and four different between-node correlation patterns using BSLMM as the imputation model in TWAS. The red dotted line represents the significance level ($\alpha=0.05$). (A) Only node has effect; (B) Only edge has effect; the results for effecting node (C) or for effecting edge (D) when both node and edge change with changing node hanging on the edge; the results for effecting node (E) or for effecting edge (F) when both node and edge change with changing node not hanging on the edge.

**

Figure S4 | Simulation results of lipid and atherosclerosis network under random effecting nodes or edges.** Type I error and power of both NeRiT and PMNT with data simulated based on lipid and atherosclerosis network under random effecting nodes or edges and four different between-node correlation patterns using DPR as the imputation model in TWAS. The red dotted line represents the significance level ($\alpha=0.05$). (A) Only node has effect; (B) Only edge has effect; the results for effecting node (C) or for effecting edge (D) when both node and edge change with changing node hanging on the edge; the results for effecting node (E) or for effecting edge (F) when both node and edge change with changing node not hanging on the edge.

**

Figure S5 | Simulation results of lipid and atherosclerosis network under fixed effecting nodes or edges.** Type I error and power of both NeRiT and PMNT with data simulated based on lipid and atherosclerosis network under fixed effecting nodes or edges and four different between-node correlation patterns using BSLMM as the imputation model in TWAS. The red dotted line represents the significance level ($\alpha=0.05$). (A) Only node has effect; (B) Only edge has effect; the results for effecting node (C) or for effecting edge (D) when both node and edge change with changing node hanging on the edge; the results for effecting node (E) or for effecting edge (F) when both node and edge change with changing node not hanging on the edge.

**

Figure S6 | Simulation results of lipid and atherosclerosis network under random effecting nodes or edges.** Type I error and power of both NeRiT and PMNT with data simulated based on lipid and atherosclerosis network under random effecting nodes or edges and four different between-node correlation patterns using BSLMM as the imputation model in TWAS. The red dotted line represents the significance level ($\alpha=0.05$). (A) Only node has effect; (B) Only edge has effect; the results for effecting node (C) or for effecting edge (D) when both node and edge change with changing node hanging on the edge; the results for effecting node (E) or for effecting edge (F) when both node and edge change with changing node not hanging on the edge.


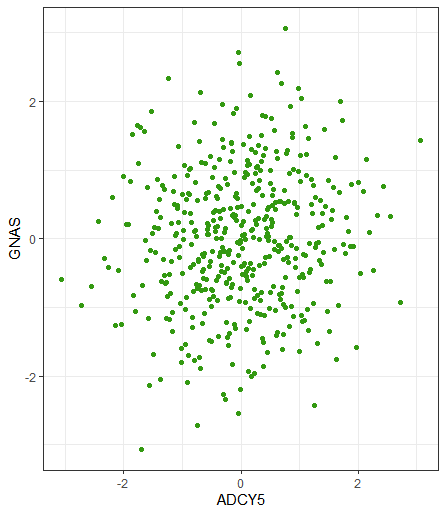


**Figure S7 |** **The scatter plots of relationship between the expression of *GNAS* and *ADCY5* in eQTL study.**

**
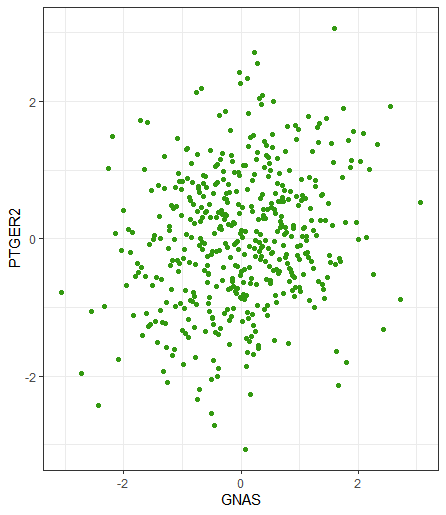
**

**Figure S8 | The scatter plots of relationship between the expression of *GNAS* and *PTGER2* in eQTL study.**

**
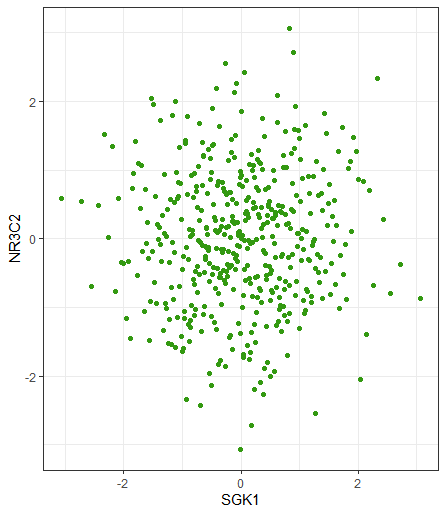
**

**Figure S9 | The scatter plots of relationship between the expression of *SGK1* and *NR3C2* in eQTL study.**

**Supplemental Tables**

Table S1 | Renin secretion network regression of both methods with *p* values in parenthesis.

|  | | **NeRiT** | **PMNT** |
| --- | --- | --- | --- |
| **SBP** | **Nodes** | *KCNMA1* ($0.008$)  *NPR1* ($0.028$) | *KCNMA1* ($0.014$)  *NPR1* ($0.029$) |
|  | **Edges** | *PRKACA-KCNMA1* ($0.028$) |  |
| **DBP** | **Nodes** | *ADRB1* ($0.0002$)  *NPR1* ($0.010$) | *ADRB1* ($0.0002$)  *NPR1* ($0.010$) |
|  | **Edges** | *PRKACA -KCNMA1* ($0.032$) |  |

Table S2 | Aldosterone-regulated sodium reabsorption network regression of both methods with *p* values in parenthesis.

|  | | **NeRiT** | **PMNT** |
| --- | --- | --- | --- |
| **SBP** | **Nodes** | *MAPK1* ($0.048$) | *MAPK1* ($0.048$) |
|  | **Edges** | *KCNJ1-NR3C2* ($0.498$) |  |
| **DBP** | **Nodes** |  |  |
|  | **Edges** | *SGK1-NEDD4L* ($0.007$) | *SGK1-NR3C2* ($0.010$) |

Table S3 | Results of the renin secretion network regression on SBP using DPR as the imputation model.

|  | **NeRiT** | | | | | **PMNT** | | | | |
| --- | --- | --- | --- | --- | --- | --- | --- | --- | --- | --- |
| Gene | Est | Std | P value | Bonferroni | FDR | Est | Std | P value | Bonferroni | FDR |
| *CREB1* | -0.0058 | 0.0018 | 0.0014 | 0.0294 | 0.0294 | -0.0056 | 0.0018 | 0.0018 | 0.0377 | 0.0377 |
| *ADRB1* | 0.0039 | 0.0018 | 0.0293 | 0.6147 | 0.3074 | 0.0040 | 0.0018 | 0.0248 | 0.5217 | 0.1761 |
| *GNAS-ADCY5* | -0.0652 | 0.0341 | 0.0554 | 1.0000 | 0.3482 | -0.0040 | 0.0018 | 0.0252 | 0.5283 | 0.1761 |
| *AQP1* | -0.0032 | 0.0018 | 0.0718 | 1.0000 | 0.3482 | -0.0033 | 0.0018 | 0.0663 | 1.0000 | 0.2786 |
| *KCNJ2* | -0.0030 | 0.0018 | 0.0915 | 1.0000 | 0.3482 | -0.0030 | 0.0018 | 0.0922 | 1.0000 | 0.3226 |
| *PRKACA-CREB1* | 0.0468 | 0.0290 | 0.1067 | 1.0000 | 0.3482 | -0.0017 | 0.0018 | 0.3570 | 1.0000 | 0.6247 |
| *CACNA1C-KCNJ2* | 0.0588 | 0.0374 | 0.1161 | 1.0000 | 0.3482 | 0.0001 | 0.0018 | 0.9410 | 1.0000 | 0.9755 |
| *ADCY5* | -0.0025 | 0.0018 | 0.1650 | 1.0000 | 0.4331 | -0.0025 | 0.0018 | 0.1692 | 1.0000 | 0.4441 |
| *GNAS-PTGER2* | 0.0309 | 0.0233 | 0.1861 | 1.0000 | 0.4343 | 0.0027 | 0.0018 | 0.1327 | 1.0000 | 0.3980 |
| *NPR1* | -0.0022 | 0.0018 | 0.2156 | 1.0000 | 0.4527 | -0.0022 | 0.0018 | 0.2128 | 1.0000 | 0.4966 |
| *ADCY5-PRKACA* | 0.0470 | 0.0411 | 0.2526 | 1.0000 | 0.4823 | -0.0001 | 0.0018 | 0.9755 | 1.0000 | 0.9755 |
| *PRKACA* | 0.0018 | 0.0018 | 0.3129 | 1.0000 | 0.5476 | 0.0018 | 0.0018 | 0.3135 | 1.0000 | 0.6247 |
| *PTGER2* | 0.0014 | 0.0018 | 0.4464 | 1.0000 | 0.7210 | 0.0017 | 0.0018 | 0.3489 | 1.0000 | 0.6247 |
| *ADORA1* | -0.0010 | 0.0018 | 0.5621 | 1.0000 | 0.7647 | -0.0010 | 0.0018 | 0.5670 | 1.0000 | 0.8437 |
| *CACNA1C* | -0.0010 | 0.0018 | 0.5971 | 1.0000 | 0.7647 | -0.0010 | 0.0018 | 0.5940 | 1.0000 | 0.8437 |
| *GNAQ* | -0.0009 | 0.0018 | 0.5998 | 1.0000 | 0.7647 | -0.0009 | 0.0018 | 0.6027 | 1.0000 | 0.8437 |
| *ADRB1-GNAS* | 0.0012 | 0.0025 | 0.6190 | 1.0000 | 0.7647 | -0.0008 | 0.0018 | 0.6593 | 1.0000 | 0.8456 |
| *GNAS* | -0.0006 | 0.0018 | 0.7253 | 1.0000 | 0.7944 | -0.0007 | 0.0018 | 0.6845 | 1.0000 | 0.8456 |
| *KCNMA1* | 0.0006 | 0.0018 | 0.7274 | 1.0000 | 0.7944 | 0.0006 | 0.0018 | 0.7311 | 1.0000 | 0.8529 |
| *PTGER2-AQP1* | 0.0070 | 0.0227 | 0.7566 | 1.0000 | 0.7944 | 0.0001 | 0.0018 | 0.9702 | 1.0000 | 0.9755 |
| *PRKACA-KCNMA1* | 0.0048 | 0.0333 | 0.8863 | 1.0000 | 0.8863 | -0.0034 | 0.0018 | 0.0587 | 1.0000 | 0.2786 |

Table S4 | Results of the renin secretion network regression on DBP using DPR as the imputation model.

|  | | **NeRiT** | | | | | | **PMNT** | | | | |
| --- | --- | --- | --- | --- | --- | --- | --- | --- | --- | --- | --- | --- |
| Gene | Est | | Std | P value | Bonferroni | FDR | Est | | Std | P value | Bonferroni | FDR |
| *ADRB1* | 0.0076 | | 0.0018 | 0.0000 | 0.0005 | 0.0005 | 0.0076 | | 0.0018 | 0.0000 | 0.0006 | 0.0006 |
| *GNAS-ADCY5* | -0.0767 | | 0.0341 | 0.0243 | 0.5093 | 0.2546 | -0.0030 | | 0.0018 | 0.1000 | 1.0000 | 0.5249 |
| *GNAS-PTGER2* | 0.0460 | | 0.0233 | 0.0489 | 1.0000 | 0.3422 | 0.0034 | | 0.0018 | 0.0593 | 1.0000 | 0.5219 |
| *CREB1* | -0.0033 | | 0.0018 | 0.0677 | 1.0000 | 0.3552 | -0.0032 | | 0.0018 | 0.0746 | 1.0000 | 0.5219 |
| *PRKACA* | 0.0025 | | 0.0018 | 0.1589 | 1.0000 | 0.6674 | 0.0025 | | 0.0018 | 0.1587 | 1.0000 | 0.6203 |
| *GNAQ* | -0.0023 | | 0.0018 | 0.1949 | 1.0000 | 0.6820 | -0.0023 | | 0.0018 | 0.1957 | 1.0000 | 0.6203 |
| *AQP1* | -0.0021 | | 0.0018 | 0.2520 | 1.0000 | 0.6987 | -0.0020 | | 0.0018 | 0.2578 | 1.0000 | 0.6766 |
| *CACNA1C-KCNJ2* | 0.0363 | | 0.0374 | 0.3328 | 1.0000 | 0.6987 | -0.0001 | | 0.0018 | 0.9468 | 1.0000 | 0.9558 |
| *KCNJ2* | -0.0017 | | 0.0018 | 0.3597 | 1.0000 | 0.6987 | -0.0016 | | 0.0018 | 0.3623 | 1.0000 | 0.8156 |
| *ADCY5* | -0.0016 | | 0.0018 | 0.3649 | 1.0000 | 0.6987 | -0.0016 | | 0.0018 | 0.3884 | 1.0000 | 0.8156 |
| *PRKACA-CREB1* | 0.0262 | | 0.0290 | 0.3660 | 1.0000 | 0.6987 | 0.0006 | | 0.0018 | 0.7530 | 1.0000 | 0.9558 |
| *ADCY5-PRKACA* | 0.0262 | | 0.0411 | 0.5237 | 1.0000 | 0.9102 | 0.0012 | | 0.0018 | 0.5220 | 1.0000 | 0.9086 |
| *ADORA1* | 0.0010 | | 0.0018 | 0.5635 | 1.0000 | 0.9102 | 0.0010 | | 0.0018 | 0.5625 | 1.0000 | 0.9086 |
| *GNAS* | -0.0009 | | 0.0018 | 0.6264 | 1.0000 | 0.9289 | -0.0009 | | 0.0018 | 0.6062 | 1.0000 | 0.9093 |
| *PTGER2* | 0.0008 | | 0.0018 | 0.6635 | 1.0000 | 0.9289 | 0.0011 | | 0.0018 | 0.5235 | 1.0000 | 0.9086 |
| *ADRB1-GNAS* | -0.0006 | | 0.0025 | 0.8207 | 1.0000 | 0.9465 | 0.0023 | | 0.0018 | 0.2068 | 1.0000 | 0.6203 |
| *CACNA1C* | 0.0004 | | 0.0018 | 0.8234 | 1.0000 | 0.9465 | 0.0004 | | 0.0018 | 0.8181 | 1.0000 | 0.9558 |
| *PTGER2-AQP1* | -0.0042 | | 0.0227 | 0.8530 | 1.0000 | 0.9465 | 0.0004 | | 0.0018 | 0.8322 | 1.0000 | 0.9558 |
| *KCNMA1* | 0.0002 | | 0.0018 | 0.9003 | 1.0000 | 0.9465 | 0.0002 | | 0.0018 | 0.8990 | 1.0000 | 0.9558 |
| *PRKACA-KCNMA1* | 0.0027 | | 0.0334 | 0.9350 | 1.0000 | 0.9465 | 0.0001 | | 0.0018 | 0.9457 | 1.0000 | 0.9558 |
| *NPR1* | 0.0001 | | 0.0018 | 0.9465 | 1.0000 | 0.9465 | 0.0001 | | 0.0018 | 0.9558 | 1.0000 | 0.9558 |

Table S5 | Results of the renin secretion network regression on SBP using BSLMM as the imputation model.

|  | | **NeRiT** | | | | | **PMNT** | | | | | |
| --- | --- | --- | --- | --- | --- | --- | --- | --- | --- | --- | --- | --- |
| Gene | Est | | Std | P value | Bonferroni | FDR | | Est | Std | P value | Bonferroni | FDR |
| *PRKACA-KCNMA1* | -0.0197 | | 0.0069 | 0.0044 | 0.0921 | 0.0822 | | -0.0018 | 0.0018 | 0.3101 | 1.0000 | 0.6513 |
| *KCNMA1* | 0.0048 | | 0.0018 | 0.0078 | 0.1644 | 0.0822 | | 0.0044 | 0.0018 | 0.0138 | 0.2892 | 0.2892 |
| *NPR1* | -0.0040 | | 0.0018 | 0.0282 | 0.5923 | 0.1974 | | -0.0039 | 0.0018 | 0.0291 | 0.6109 | 0.3055 |
| *CACNA1C-KCNJ2* | 0.0693 | | 0.0370 | 0.0612 | 1.0000 | 0.3214 | | -0.0008 | 0.0018 | 0.6694 | 1.0000 | 0.8668 |
| *ADRB1* | 0.0030 | | 0.0018 | 0.1026 | 1.0000 | 0.4308 | | 0.0028 | 0.0018 | 0.1161 | 1.0000 | 0.6058 |
| *GNAS* | -0.0028 | | 0.0018 | 0.1249 | 1.0000 | 0.4370 | | -0.0028 | 0.0018 | 0.1151 | 1.0000 | 0.6058 |
| *AQP1* | -0.0026 | | 0.0018 | 0.1478 | 1.0000 | 0.4434 | | -0.0026 | 0.0018 | 0.1494 | 1.0000 | 0.6058 |
| *KCNJ2* | -0.0025 | | 0.0018 | 0.1730 | 1.0000 | 0.4540 | | -0.0025 | 0.0018 | 0.1731 | 1.0000 | 0.6058 |
| *GNAS-PTGER2* | 0.0281 | | 0.0242 | 0.2452 | 1.0000 | 0.5466 | | 0.0019 | 0.0018 | 0.2872 | 1.0000 | 0.6513 |
| *ADCY5-PRKACA* | 0.0350 | | 0.0311 | 0.2613 | 1.0000 | 0.5466 | | -0.0023 | 0.0018 | 0.2125 | 1.0000 | 0.6375 |
| *ADCY5* | -0.0019 | | 0.0018 | 0.2863 | 1.0000 | 0.5466 | | -0.0021 | 0.0018 | 0.2537 | 1.0000 | 0.6513 |
| *ADRB1-GNAS* | -0.0135 | | 0.0169 | 0.4221 | 1.0000 | 0.7386 | | -0.0007 | 0.0018 | 0.6769 | 1.0000 | 0.8668 |
| *PTGER2* | 0.0013 | | 0.0018 | 0.4838 | 1.0000 | 0.7815 | | 0.0014 | 0.0018 | 0.4211 | 1.0000 | 0.8040 |
| *CREB1* | -0.0008 | | 0.0018 | 0.6459 | 1.0000 | 0.9591 | | -0.0008 | 0.0018 | 0.6486 | 1.0000 | 0.8668 |
| *PRKACA* | -0.0007 | | 0.0018 | 0.7133 | 1.0000 | 0.9591 | | -0.0006 | 0.0018 | 0.7273 | 1.0000 | 0.8668 |
| *CACNA1C* | 0.0005 | | 0.0018 | 0.7681 | 1.0000 | 0.9591 | | 0.0006 | 0.0018 | 0.7429 | 1.0000 | 0.8668 |
| *PRKACA-CREB1* | -0.0079 | | 0.0280 | 0.7765 | 1.0000 | 0.9591 | | -0.0001 | 0.0018 | 0.9407 | 1.0000 | 0.9827 |
| *GNAS-ADCY5* | -0.0049 | | 0.0295 | 0.8685 | 1.0000 | 0.9782 | | 0.0009 | 0.0018 | 0.6186 | 1.0000 | 0.8668 |
| *PTGER2-AQP1* | 0.0040 | | 0.0281 | 0.8874 | 1.0000 | 0.9782 | | 0.0012 | 0.0018 | 0.5208 | 1.0000 | 0.8668 |
| *ADORA1* | -0.0001 | | 0.0018 | 0.9382 | 1.0000 | 0.9782 | | -0.0001 | 0.0018 | 0.9382 | 1.0000 | 0.9827 |
| *GNAQ* | 0.0000 | | 0.0018 | 0.9782 | 1.0000 | 0.9782 | | 0.0000 | 0.0018 | 0.9827 | 1.0000 | 0.9827 |

Table S6 | Results of the renin secretion network regression on DBP using BSLMM as the imputation model.

|  | | **NeRiT** | | | | | **PMNT** | | | | | |  |
| --- | --- | --- | --- | --- | --- | --- | --- | --- | --- | --- | --- | --- | --- |
| Gene | | Est | | Std | P value | Bonferroni | FDR | | Est | Std | P value | Bonferroni | FDR |
| *ADRB1* | | 0.0068 | | 0.0018 | 0.0002 | 0.0039 | 0.0039 | | 0.0066 | 0.0018 | 0.0002 | 0.0049 | 0.0049 |
| *NPR1* | | -0.0046 | | 0.0018 | 0.0099 | 0.2084 | 0.1042 | | -0.0046 | 0.0018 | 0.0100 | 0.2096 | 0.1048 |
| *PRKACA-KCNMA1* | | -0.0148 | | 0.0069 | 0.0320 | 0.6710 | 0.2237 | | 0.0014 | 0.0018 | 0.4391 | 1.0000 | 0.9769 |
| *KCNMA1* | | 0.0035 | | 0.0018 | 0.0546 | 1.0000 | 0.2866 | | 0.0032 | 0.0018 | 0.0772 | 1.0000 | 0.5402 |
| *GNAS-ADCY5* | | -0.0367 | | 0.0295 | 0.2143 | 1.0000 | 0.7806 | | 0.0004 | 0.0018 | 0.8319 | 1.0000 | 0.9769 |
| *ADCY5-PRKACA* | | 0.0339 | | 0.0311 | 0.2768 | 1.0000 | 0.7806 | | -0.0006 | 0.0018 | 0.7330 | 1.0000 | 0.9769 |
| *GNAQ* | | -0.0017 | | 0.0018 | 0.3340 | 1.0000 | 0.7806 | | -0.0017 | 0.0018 | 0.3334 | 1.0000 | 0.9769 |
| *CACNA1C-KCNJ2* | | 0.0357 | | 0.0370 | 0.3354 | 1.0000 | 0.7806 | | 0.0016 | 0.0018 | 0.3865 | 1.0000 | 0.9769 |
| *ADCY5* | | -0.0017 | | 0.0018 | 0.3473 | 1.0000 | 0.7806 | | -0.0017 | 0.0018 | 0.3375 | 1.0000 | 0.9769 |
| *AQP1* | | -0.0016 | | 0.0018 | 0.3868 | 1.0000 | 0.7806 | | -0.0015 | 0.0018 | 0.3946 | 1.0000 | 0.9769 |
| *ADRB1-GNAS* | | -0.0139 | | 0.0169 | 0.4089 | 1.0000 | 0.7806 | | 0.0013 | 0.0018 | 0.4686 | 1.0000 | 0.9769 |
| *GNAS-PTGER2* | | 0.0149 | | 0.0242 | 0.5373 | 1.0000 | 0.8797 | | 0.0026 | 0.0018 | 0.1465 | 1.0000 | 0.7690 |
| *GNAS* | | -0.0011 | | 0.0018 | 0.5446 | 1.0000 | 0.8797 | | -0.0009 | 0.0018 | 0.6051 | 1.0000 | 0.9769 |
| *PTGER2* | | 0.0007 | | 0.0018 | 0.6975 | 1.0000 | 0.9778 | | 0.0008 | 0.0018 | 0.6678 | 1.0000 | 0.9769 |
| *CREB1* | | 0.0006 | | 0.0018 | 0.7435 | 1.0000 | 0.9778 | | 0.0006 | 0.0018 | 0.7603 | 1.0000 | 0.9769 |
| *PRKACA* | | -0.0005 | | 0.0018 | 0.7734 | 1.0000 | 0.9778 | | -0.0005 | 0.0018 | 0.7607 | 1.0000 | 0.9769 |
| *PRKACA-CREB1* | | 0.0063 | | 0.0280 | 0.8208 | 1.0000 | 0.9778 | | -0.0001 | 0.0018 | 0.9443 | 1.0000 | 0.9769 |
| *ADORA1* | | 0.0003 | | 0.0018 | 0.8524 | 1.0000 | 0.9778 | | 0.0003 | 0.0018 | 0.8557 | 1.0000 | 0.9769 |
| *KCNJ2* | | -0.0003 | | 0.0018 | 0.8847 | 1.0000 | 0.9778 | | -0.0003 | 0.0018 | 0.8863 | 1.0000 | 0.9769 |
| *CACNA1C* | | 0.0001 | | 0.0018 | 0.9395 | 1.0000 | 0.9811 | | 0.0002 | 0.0018 | 0.9225 | 1.0000 | 0.9769 |
| *PTGER2-AQP1* | | -0.0007 | | 0.0281 | 0.9811 | 1.0000 | 0.9811 | | 0.0001 | 0.0018 | 0.9769 | 1.0000 | 0.9769 |

Table S7 | Results of the aldosterone-regulated sodium reabsorption network regression on SBP using DPR as the imputation model.

|  | | **NeRiT** | | | | | **PMNT** | | | | | |  |
| --- | --- | --- | --- | --- | --- | --- | --- | --- | --- | --- | --- | --- | --- |
| Gene | | Est | | Std | P value | Bonferroni | FDR | | Est | Std | P value | Bonferroni | FDR |
| *IGF1* | | 0.0042 | | 0.0018 | 0.0200 | 0.3806 | 0.2232 | | 0.0041 | 0.0018 | 0.0213 | 0.4041 | 0.2267 |
| *MAPK1* | | -0.0040 | | 0.0018 | 0.0277 | 0.5254 | 0.2232 | | -0.0040 | 0.0018 | 0.0272 | 0.5175 | 0.2267 |
| *SLC9A3R2* | | -0.0037 | | 0.0018 | 0.0394 | 0.7488 | 0.2232 | | -0.0037 | 0.0018 | 0.0399 | 0.7589 | 0.2267 |
| *IRS1* | | 0.0036 | | 0.0018 | 0.0470 | 0.8928 | 0.2232 | | 0.0036 | 0.0018 | 0.0477 | 0.9068 | 0.2267 |
| *SGK1-NR3C2* | | 0.0641 | | 0.0343 | 0.0614 | 1.0000 | 0.2333 | | -0.0022 | 0.0018 | 0.2307 | 1.0000 | 0.5478 |
| *SFN* | | -0.0031 | | 0.0018 | 0.0891 | 1.0000 | 0.2821 | | -0.0031 | 0.0018 | 0.0898 | 1.0000 | 0.2844 |
| *NEDD4L* | | -0.0029 | | 0.0018 | 0.1088 | 1.0000 | 0.2953 | | -0.0029 | 0.0018 | 0.1115 | 1.0000 | 0.3026 |
| *IRS1-INSR* | | -0.0278 | | 0.0192 | 0.1475 | 1.0000 | 0.3504 | | 0.0001 | 0.0018 | 0.9369 | 1.0000 | 0.9972 |
| *PIK3CA-IRS1* | | 0.0248 | | 0.0192 | 0.1972 | 1.0000 | 0.4163 | | -0.0004 | 0.0018 | 0.8182 | 1.0000 | 0.9972 |
| *PRKCA* | | 0.0020 | | 0.0018 | 0.2752 | 1.0000 | 0.5229 | | 0.0020 | 0.0018 | 0.2711 | 1.0000 | 0.5724 |
| *PIK3CA* | | 0.0016 | | 0.0018 | 0.3727 | 1.0000 | 0.6437 | | 0.0017 | 0.0018 | 0.3430 | 1.0000 | 0.6517 |
| *INSR-IGF1* | | 0.0266 | | 0.0352 | 0.4506 | 1.0000 | 0.7134 | | -0.0003 | 0.0018 | 0.8593 | 1.0000 | 0.9972 |
| *KCNJ1-SGK1* | | 0.0226 | | 0.0344 | 0.5112 | 1.0000 | 0.7472 | | -0.0001 | 0.0018 | 0.9635 | 1.0000 | 0.9972 |
| *SGK1-NEDD4L* | | -0.0198 | | 0.0359 | 0.5816 | 1.0000 | 0.7893 | | 0.0000 | 0.0018 | 0.9972 | 1.0000 | 0.9972 |
| *NR3C2* | | 0.0008 | | 0.0018 | 0.6723 | 1.0000 | 0.8279 | | 0.0008 | 0.0018 | 0.6402 | 1.0000 | 0.9972 |
| *KCNJ1* | | -0.0007 | | 0.0018 | 0.6971 | 1.0000 | 0.8279 | | -0.0007 | 0.0018 | 0.7005 | 1.0000 | 0.9972 |
| *SGK1* | | 0.0004 | | 0.0018 | 0.8158 | 1.0000 | 0.8613 | | 0.0004 | 0.0018 | 0.8038 | 1.0000 | 0.9972 |
| *NEDD4L-SFN* | | -0.0098 | | 0.0423 | 0.8160 | 1.0000 | 0.8613 | | -0.0032 | 0.0018 | 0.0779 | 1.0000 | 0.2844 |
| *INSR* | | 0.0001 | | 0.0018 | 0.9338 | 1.0000 | 0.9338 | | 0.0001 | 0.0018 | 0.9440 | 1.0000 | 0.9972 |

Table S8 | Results of the aldosterone-regulated sodium reabsorption network regression on DBP using DPR as the imputation model.

|  | | **NeRiT** | | | | | **PMNT** | | | | | |  |
| --- | --- | --- | --- | --- | --- | --- | --- | --- | --- | --- | --- | --- | --- |
| Gene | | Est | | Std | P value | Bonferroni | FDR | | Est | Std | P value | Bonferroni | FDR |
| *NEDD4L* | | -0.0045 | | 0.0018 | 0.0128 | 0.2435 | 0.2435 | | -0.0045 | 0.0018 | 0.0131 | 0.2483 | 0.2483 |
| *SGK1-NR3C2* | | 0.0690 | | 0.0343 | 0.0441 | 0.8373 | 0.4187 | | -0.0025 | 0.0018 | 0.1639 | 1.0000 | 0.5248 |
| *KCNJ1* | | 0.0032 | | 0.0018 | 0.0723 | 1.0000 | 0.4577 | | 0.0032 | 0.0018 | 0.0721 | 1.0000 | 0.4569 |
| *SFN* | | -0.0025 | | 0.0018 | 0.1655 | 1.0000 | 0.4718 | | -0.0023 | 0.0018 | 0.1933 | 1.0000 | 0.5248 |
| *SLC9A3R2* | | -0.0024 | | 0.0018 | 0.1785 | 1.0000 | 0.4718 | | -0.0024 | 0.0018 | 0.1804 | 1.0000 | 0.5248 |
| *IGF1* | | 0.0024 | | 0.0018 | 0.1787 | 1.0000 | 0.4718 | | 0.0024 | 0.0018 | 0.1900 | 1.0000 | 0.5248 |
| *NEDD4L-SFN* | | -0.0560 | | 0.0423 | 0.1852 | 1.0000 | 0.4718 | | -0.0035 | 0.0018 | 0.0531 | 1.0000 | 0.4569 |
| *INSR-IGF1* | | 0.0416 | | 0.0352 | 0.2371 | 1.0000 | 0.4718 | | -0.0002 | 0.0018 | 0.8981 | 1.0000 | 0.9057 |
| *PRKCA* | | 0.0021 | | 0.0018 | 0.2530 | 1.0000 | 0.4718 | | 0.0021 | 0.0018 | 0.2472 | 1.0000 | 0.5471 |
| *SGK1* | | 0.0020 | | 0.0018 | 0.2681 | 1.0000 | 0.4718 | | 0.0020 | 0.0018 | 0.2592 | 1.0000 | 0.5471 |
| *IRS1* | | 0.0019 | | 0.0018 | 0.2832 | 1.0000 | 0.4718 | | 0.0018 | 0.0018 | 0.3151 | 1.0000 | 0.5987 |
| *PIK3CA-IRS1* | | -0.0200 | | 0.0192 | 0.2980 | 1.0000 | 0.4718 | | 0.0008 | 0.0018 | 0.6608 | 1.0000 | 0.7847 |
| *IRS1-INSR* | | 0.0139 | | 0.0192 | 0.4692 | 1.0000 | 0.6426 | | 0.0009 | 0.0018 | 0.6365 | 1.0000 | 0.7847 |
| *MAPK1* | | -0.0013 | | 0.0018 | 0.4735 | 1.0000 | 0.6426 | | -0.0013 | 0.0018 | 0.4652 | 1.0000 | 0.7725 |
| *NR3C2* | | -0.0010 | | 0.0018 | 0.5681 | 1.0000 | 0.7196 | | -0.0010 | 0.0018 | 0.5880 | 1.0000 | 0.7847 |
| *INSR* | | -0.0008 | | 0.0018 | 0.6417 | 1.0000 | 0.7620 | | -0.0008 | 0.0018 | 0.6529 | 1.0000 | 0.7847 |
| *SGK1-NEDD4L* | | 0.0118 | | 0.0359 | 0.7421 | 1.0000 | 0.8294 | | 0.0013 | 0.0018 | 0.4879 | 1.0000 | 0.7725 |
| *KCNJ1-SGK1* | | -0.0089 | | 0.0344 | 0.7961 | 1.0000 | 0.8404 | | 0.0006 | 0.0018 | 0.7454 | 1.0000 | 0.8331 |
| *PIK3CA* | | 0.0003 | | 0.0018 | 0.8765 | 1.0000 | 0.8765 | | 0.0002 | 0.0018 | 0.9057 | 1.0000 | 0.9057 |

Table S9 | Results of the aldosterone-regulated sodium reabsorption network regression on SBP using BSLMM as the imputation model.

|  | | **NeRiT** | | | | | **PMNT** | | | | | |  |
| --- | --- | --- | --- | --- | --- | --- | --- | --- | --- | --- | --- | --- | --- |
| Gene | | Est | | Std | P value | Bonferroni | FDR | | Est | Std | P value | Bonferroni | FDR |
| *MAPK1* | | -0.0036 | | 0.0018 | 0.0482 | 0.9154 | 0.3699 | | -0.0036 | 0.0018 | 0.0482 | 0.9167 | 0.5382 |
| *KCNJ1-SGK1* | | 0.0661 | | 0.0337 | 0.0498 | 0.9462 | 0.3699 | | 0.0017 | 0.0018 | 0.3412 | 1.0000 | 0.7438 |
| *IRS1* | | 0.0034 | | 0.0018 | 0.0584 | 1.0000 | 0.3699 | | 0.0034 | 0.0018 | 0.0567 | 1.0000 | 0.5382 |
| *SGK1-NEDD4L* | | -0.0514 | | 0.0350 | 0.1422 | 1.0000 | 0.6378 | | 0.0028 | 0.0018 | 0.1159 | 1.0000 | 0.5506 |
| *NEDD4L* | | -0.0025 | | 0.0018 | 0.1679 | 1.0000 | 0.6378 | | -0.0025 | 0.0018 | 0.1662 | 1.0000 | 0.6317 |
| *NR3C2* | | 0.0020 | | 0.0018 | 0.2762 | 1.0000 | 0.7476 | | 0.0022 | 0.0018 | 0.2221 | 1.0000 | 0.7034 |
| *SGK1-NR3C2* | | -0.0266 | | 0.0259 | 0.3054 | 1.0000 | 0.7476 | | -0.0030 | 0.0018 | 0.0951 | 1.0000 | 0.5506 |
| *INSR-IGF1* | | -0.0288 | | 0.0310 | 0.3522 | 1.0000 | 0.7476 | | 0.0017 | 0.0018 | 0.3404 | 1.0000 | 0.7438 |
| *SLC9A3R2* | | -0.0017 | | 0.0018 | 0.3541 | 1.0000 | 0.7476 | | -0.0017 | 0.0018 | 0.3523 | 1.0000 | 0.7438 |
| *SGK1* | | 0.0014 | | 0.0018 | 0.4311 | 1.0000 | 0.7698 | | 0.0014 | 0.0018 | 0.4398 | 1.0000 | 0.7842 |
| *INSR* | | -0.0012 | | 0.0018 | 0.5052 | 1.0000 | 0.7698 | | -0.0012 | 0.0018 | 0.5227 | 1.0000 | 0.7842 |
| *PRKCA* | | 0.0011 | | 0.0018 | 0.5291 | 1.0000 | 0.7698 | | 0.0011 | 0.0018 | 0.5327 | 1.0000 | 0.7842 |
| *NEDD4L-SFN* | | 0.0112 | | 0.0218 | 0.6085 | 1.0000 | 0.7698 | | -0.0007 | 0.0018 | 0.7148 | 1.0000 | 0.8292 |
| *IGF1* | | 0.0009 | | 0.0018 | 0.6095 | 1.0000 | 0.7698 | | 0.0009 | 0.0018 | 0.6191 | 1.0000 | 0.7842 |
| *SFN* | | -0.0009 | | 0.0018 | 0.6325 | 1.0000 | 0.7698 | | -0.0009 | 0.0018 | 0.6155 | 1.0000 | 0.7842 |
| *PIK3CA-IRS1* | | 0.0141 | | 0.0310 | 0.6483 | 1.0000 | 0.7698 | | -0.0010 | 0.0018 | 0.5751 | 1.0000 | 0.7842 |
| *PIK3CA* | | 0.0005 | | 0.0018 | 0.7897 | 1.0000 | 0.8826 | | 0.0005 | 0.0018 | 0.7856 | 1.0000 | 0.8292 |
| *KCNJ1* | | 0.0000 | | 0.0018 | 0.9899 | 1.0000 | 0.9988 | | -0.0001 | 0.0018 | 0.9711 | 1.0000 | 0.9711 |
| *IRS1-INSR* | | 0.0000 | | 0.0335 | 0.9988 | 1.0000 | 0.9988 | | -0.0005 | 0.0018 | 0.7756 | 1.0000 | 0.8292 |

Table S10 | Results of the aldosterone-regulated sodium reabsorption network regression on DBP using BSLMM as the imputation model.

|  | | **NeRiT** | | | | | **PMNT** | | | | | |  |
| --- | --- | --- | --- | --- | --- | --- | --- | --- | --- | --- | --- | --- | --- |
| Gene | | Est | | Std | P value | Bonferroni | FDR | | Est | Std | P value | Bonferroni | FDR |
| *SGK1-NEDD4L* | | -0.0953 | | 0.0350 | 0.0065 | 0.1235 | 0.1235 | | 0.0020 | 0.0018 | 0.2697 | 1.0000 | 0.5897 |
| *INSR* | | -0.0029 | | 0.0018 | 0.1019 | 1.0000 | 0.6501 | | -0.0030 | 0.0018 | 0.0968 | 1.0000 | 0.5897 |
| *KCNJ1* | | 0.0029 | | 0.0018 | 0.1026 | 1.0000 | 0.6501 | | 0.0029 | 0.0018 | 0.1079 | 1.0000 | 0.5897 |
| *KCNJ1-SGK1* | | 0.0460 | | 0.0337 | 0.1719 | 1.0000 | 0.7243 | | 0.0018 | 0.0018 | 0.3104 | 1.0000 | 0.5897 |
| *NEDD4L* | | -0.0023 | | 0.0018 | 0.2057 | 1.0000 | 0.7243 | | -0.0023 | 0.0018 | 0.2048 | 1.0000 | 0.5897 |
| *PRKCA* | | 0.0020 | | 0.0018 | 0.2606 | 1.0000 | 0.7243 | | 0.0020 | 0.0018 | 0.2595 | 1.0000 | 0.5897 |
| *SLC9A3R2* | | -0.0020 | | 0.0018 | 0.2700 | 1.0000 | 0.7243 | | -0.0020 | 0.0018 | 0.2694 | 1.0000 | 0.5897 |
| *SFN* | | -0.0018 | | 0.0018 | 0.3050 | 1.0000 | 0.7243 | | -0.0019 | 0.0018 | 0.2866 | 1.0000 | 0.5897 |
| *NEDD4L-SFN* | | 0.0192 | | 0.0218 | 0.3787 | 1.0000 | 0.7897 | | -0.0016 | 0.0018 | 0.3696 | 1.0000 | 0.6384 |
| *IRS1-INSR* | | 0.0272 | | 0.0335 | 0.4156 | 1.0000 | 0.7897 | | 0.0002 | 0.0018 | 0.9256 | 1.0000 | 0.9256 |
| *MAPK1* | | -0.0011 | | 0.0018 | 0.5444 | 1.0000 | 0.8994 | | -0.0011 | 0.0018 | 0.5500 | 1.0000 | 0.8709 |
| *IRS1* | | 0.0007 | | 0.0018 | 0.6875 | 1.0000 | 0.8994 | | 0.0008 | 0.0018 | 0.6711 | 1.0000 | 0.9003 |
| *INSR-IGF1* | | -0.0111 | | 0.0310 | 0.7197 | 1.0000 | 0.8994 | | 0.0004 | 0.0018 | 0.8147 | 1.0000 | 0.9003 |
| *NR3C2* | | 0.0006 | | 0.0018 | 0.7617 | 1.0000 | 0.8994 | | 0.0006 | 0.0018 | 0.7545 | 1.0000 | 0.9003 |
| *PIK3CA-IRS1* | | 0.0086 | | 0.0310 | 0.7817 | 1.0000 | 0.8994 | | -0.0022 | 0.0018 | 0.2126 | 1.0000 | 0.5897 |
| *SGK1* | | 0.0005 | | 0.0018 | 0.7830 | 1.0000 | 0.8994 | | 0.0004 | 0.0018 | 0.8087 | 1.0000 | 0.9003 |
| *PIK3CA* | | 0.0003 | | 0.0018 | 0.8508 | 1.0000 | 0.8994 | | 0.0003 | 0.0018 | 0.8465 | 1.0000 | 0.9003 |
| *IGF1* | | -0.0003 | | 0.0018 | 0.8521 | 1.0000 | 0.8994 | | -0.0003 | 0.0018 | 0.8529 | 1.0000 | 0.9003 |
| *SGK1-NR3C2* | | -0.0003 | | 0.0259 | 0.9907 | 1.0000 | 0.9907 | | -0.0046 | 0.0018 | 0.0103 | 0.1957 | 0.1957 |
